# Radiation and chemotherapy variable response induced by tumor cell hypoxia: impact of radiation dose, anticancer drug, and type of cancer

**DOI:** 10.1007/s00411-022-00974-6

**Published:** 2022-04-09

**Authors:** Ayman M. Ibrahim, Soad Nady, Medhat W. Shafaa, Magdy M. Khalil

**Affiliations:** 1 Medical Biophysics, Department of Physics, Faculty of Science, Helwan University, Cairo, Egypt; 2 Immunology Laboratory, Department of Zoology and Entomology, Faculty of Science, Helwan University, Cairo, Egypt

**Keywords:** Cancer cell lines, Hypoxia, HIF-1α, Chemoradiotherapy

## Abstract

Hypoxia is a condition in which proliferating tumor cells are deprived of oxygen due to limited blood supply from abnormal tumor microvasculature. This study aimed to investigate the molecular changes that occur in tumor cell hypoxia with special emphasis placed on the efficacy of chemotherapeutic and radiation-related effects. Four commercially available chemotherapeutic agents: cisplatin, cyclophosphamide, doxorubicin, and 5-fluorouracil, were tested for their cytotoxic activity on the cancer cell lines PC3 (prostate), HepG2 (liver), and MCF-7 (breast). Tumor cell lines under hypoxia were treated with both IC_50_ concentrations of the different chemotherapeutic agents and irradiated with 5 and 10 Gy using a ^137^Cs gamma source. Hypoxia-inducible factor-1α (HIF-1α) protein levels were examined using an ELISA assay. Hypoxic cells showed a significant change in cell viability to all chemotherapeutic agents in comparison to normoxic controls. HepG2 cells were more resistant to the cytotoxic drug doxorubicin compared to other cancer cell lines. The flow cytometric analysis showed that hypoxic cells have lower levels of total apoptotic cell populations (early and late apoptosis) compared to normoxic cells suggesting decreased hypoxia-induced apoptosis in cancer cells. The highest reduction in HIF-1α level was observed in the MCF-7 cell line (95.5%) in response to the doxorubicin treatment combined with 10 Gy irradiation of cells. Chemoradiotherapy could result in minimal as well as a high reduction of HIF-1α based on cell type, type of chemotherapy, and amount of ionizing radiation. This study highlights future research work to optimize a combined chemoradiotherapeutic regime in individual cancer cell hypoxia.

## Introduction

Cancer is a known worldwide threat responsible for about 7.6 million deaths per year, and it is predicted to reach 13.1 million by 2030 (WHO 2017). Factors related to the microenvironment play decisive roles in cancer initiation, progression, metastasis, and chemo-radioresistance. An example of such factors is hypoxia which is considered as one of the potential cancer hallmarks, especially in solid tumors. It occurs due to abnormal vascularization and rapid proliferation of tumors that result in fluctuation of oxygen supply throughout the growing tumor mass (Brahimi-Horn et al. 2007).

Hypoxia is defined as a loss of oxygen in tissues and can occur in tumors due to limited perfusion of blood vessels (acute hypoxia) or because of limited oxygen diffusion and its consumption by tumor cells (chronic hypoxia) that leads to hypoxic regions at distances of approximately 100 to 200 µm from functional blood vessels (Brown and Wilson 2004).

Hypoxia enhances the ability of tumors to metastasize and increases their aggressiveness and resistance to apoptosis (Muz et al. 2015; Wilson and Hay 2011). It impacts significantly on radio-chemo-resistance, resulting in poor prognosis in patients with severe hypoxic neoplastic tumors (Doktorova et al. 2015). During radiotherapy, the interaction between oxygen and radiation results in the production of radical oxygen species (ROS), which cause DNA damage, thereby promoting apoptosis. On the other hand, hypoxic areas in large tumors are less susceptible to radiotherapy because of low levels of ROS (Bindra et al. 2007).

Chemo-resistance in hypoxic tumors is largely a consequence of the failure of anti-proliferative drugs to target cancer cells that have undergone hypoxia-induced reversible quiescence (Mistry et al. 2017). Furthermore, the limitations of diffusion and perfusion cause tumor hypoxia to reduce drug delivery to these regions (Huxham et al. 2004). Since the cytotoxic effect of cytostatic drugs is greater in rapidly dividing cells, the slow proliferating tumor cells far away from blood vessels are less sensitive to chemotherapy (Strese et al. 2013).

Hypoxia selects for cells with low expression of p53, and consequently, p53-induced apoptosis is reduced in hypoxic cells (Graeber et al. 1996). In normoxic surroundings, DNA injuries caused by some anticancer drugs are more permanent, whereas higher rates of restoration occur within hypoxic surroundings (Brown and Wilson 2004). Another correlation between hypoxia and chemotherapy resistance is the up-regulation of multidrug-resistance (MDR) genes and overexpression of the gene product P-glycoprotein (P-gp), known to be involved in multidrug resistance (Wartenberg et al. 2003).

Hypoxia-inducible factor 1 (HIF-1) is the heterodimer protein of two subunits: HIF-1α and HIF-1β transcriptional factor. Each contains helix–loop-(HLS-) PER-ARNT-SIM (HLS PAS) domains that facilitate DNA binding and heterodimerization. The beta subunit can also be referred to as the aryl hydrocarbon receptor nuclear translocator (ARNT). The alpha subunit is sensitive to oxygen, whereas the beta subunit (HIF-1β) is oxygen-dependent (Balamurugan 2016).

HIF-1 can essentially be interpreted as a messenger migrating towards the nucleus to activate transcription responses to hypoxia. HIF-1 has been involved in gene regulation involving metastasis, growth, tumorigenesis, angiogenesis, and invasion. The vascular endothelial growth factor (VEGF) is an example of a HIF-1 target gene in which its expression is induced by hypoxia (Akanji et al. 2019). Erythropoietin, the key regulator of red blood cell production is also one of the many target genes activated by HIF-1 under hypoxia which had helped to decipher HIF-1 activation and cellular oxygen sensing (Locatelli et al. 2017).

Despite the relatively large amount of data regarding tumor hypoxia, there might be some lack of knowledge on how the combined chemoradiotherapy could be effective in reducing the cellular concentrations of HIF-1 alpha especially when attempts are made to compare the conventionally used chemotherapeutic drugs versus specific tumor types.

This study aimed to elucidate the impact of chemoradiotherapy on different cancer cell lines, including prostate, breast, and liver under hypoxic conditions with emphasis placed on radiation dose, different chemotherapeutic agents as well as type of cancer.

## Materials and methods

### Chemotherapeutic agents

Four commercially available and clinically relevant chemotherapeutic agents, namely cisplatin, cyclophosphamide, doxorubicin, and 5-fluorouracil, were used in the study. All compounds were in liquid form and purchased from a local supplier (El-Ezaby Pharmacy Inc, Egypt) with optimal storing condition of 4 °C.

### Cell lines and culture condition

Three human cancer cell lines were used in this study originating from prostate cancer (PC3 ATCC^®^ CRL-1435^™^), liver cancer (HepG2 ATCC^®^ HB-8065^™^), and breast cancer (MCF-7 ATCC^®^ HTB-22^™^). PC3, HepG2, and MCF-7 cells were obtained from laboratory of the Tissue Culture Department of the Holding Company for Biological Products and Vaccines (VACSERA, Dokki, Egypt).

The cells were cultured at 37 °C under 5% carbon dioxide and 95% humidity in complete RPMI-1640 medium supplemented with 10% heat-inactivated fetal bovine serum (FBS), 1% antibiotic–gentamicin solution, and 1% sodium pyruvate. Cells were grown in 75 cm^2^ cell culture flasks to 80% confluence and harvested, counted, and washed with phosphate-buffered saline (PBS). All media and supplies were purchased from Lonza, Belgium.

### Hypoxia induction

Normoxic cells were maintained at 37 °C in a humidified incubator containing 21% O_2_, 5% CO_2_ in the air. To induce hypoxia; cell culture plates were placed in an acryl chamber (in-house made) and then flushed with a mixture of 1% O_2_, 5% CO_2_, and 94% N_2_ for 24 h. During hypoxia induction, the chamber was gently inflated leaving a moderate pressure over the plates with proper sealing to avoid any sort of leaking. The temperature was normally kept at 37 °C.

**Fig. 1:**
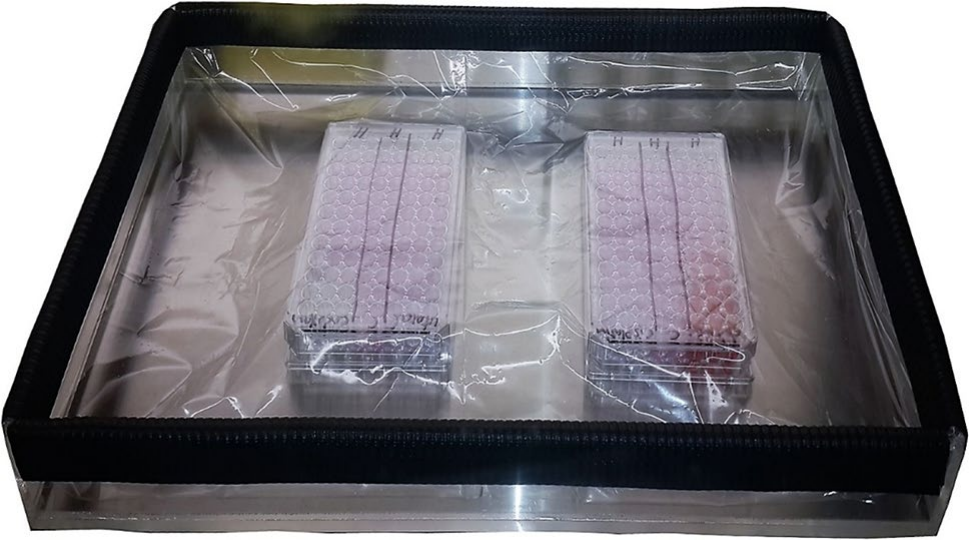
A photograph of an acrylic chamber covered with a plastic pouch. The chamber can be filled with mixed gases through a narrow hole made in one side of the chamber. The side dimensions of the chamber are 30 × 30 cm

Figure 1 shows the chamber with a plastic cover and two 96-well cell culture plates inserted inside. The black frame around the chamber was tight to prevent any leakage of the gas and maintain constant experimental conditions throughout cell incubation. This model was similar to previous reports that showed an acceptable performance in hypoxia induction (Saxena et al. 2020).

### Assessment of cell cytotoxicity by MTT assay

The cell lines PC3, HepG2, and MCF-7 were rinsed with PBS, trypsinized, and re-suspended in complete RPMI-1640 culture medium, then cells were counted using a hemocytometer chamber by Trypan blue exclusion technique (Bellamakondi et al. 2014).

For chemosensitivity studies, the cells were cultured in 96-multiwell plate at a density of 10^4^ cells/well for 24 h before treatment with the chemotherapeutic agent to allow attachment of the cells to the wall of the plate. After 24 h incubation, the medium was removed from each well and replaced with a fresh cell culture medium containing the tested compounds at different concentrations using a two-fold serial dilution method. The experiment was carried out in triplicate wells for each drug for each individual dose.

Cell monolayers were then incubated with different concentrations of chemotherapeutic agents for 48 h at 37 °C incubator supplied with 5% CO_2_ under normoxic and hypoxic conditions. The drug-containing medium was then removed, and the cells were washed twice with PBS. A volume of 50 µl of yellow methyl thiazolyl tetrazolium salt MTT 3-(4,5-dimethylthiazol-2-yl)-2,5-diphenyltetrazolium bromide (0.5 mg/ml) was immediately added to each well, and the plates were incubated for 4 h at 37 °C under normoxic and hypoxic conditions. Then the medium was removed, and 50 µl dimethyl sulfoxide (DMSO) was added to dissolve the formazan crystals.

The absorbance of the resulting color was measured using an ELISA plate reader (Boster Immunoleader, USA) at 490 nm to estimate the percentage of viable cells. Cell survival was determined as the true absorbance of the treated wells divided by the controls and expressed as a percentage. The correlation between cell viability and drug concentration was plotted to get the viability curve of each cancer cell line versus a particular drug and to determine the drug concentration that inhibits cell growth by 50% (half-maximal inhibitory concentration; IC_50_). The dose–response curve was fitted using the non-linear Boltzmann formula, *Y* = *A*_2_ + (*A*_1_−*A*_2_)/1 + e^(x−x^_°_^)/dx^ where *A*_1_ is the initial value, *A*_2_ is the final value, *x*_0_ is the center, and dx is the time constant, so that standard analytical derivation of IC_50_ can be computed.

The effect of hypoxia on the activity of drugs was expressed as the hypoxia/normoxia ratio (HNR), which is defined as the ratio of IC_50_ values under hypoxic to normoxic conditions.

### Detection of apoptosis by flow cytometry

The IC_50_ concentration of the four tested chemotherapeutic agents determined by the MTT assay was used to treat the three different cancer cell lines at a density of 1 × 10^6^ cells per 25 cm^2^ culture flasks for 24 h under normoxic and hypoxic conditions. Then, cells were trypsinized and collected by centrifugation (2000 rpm for 10 min) and removal of supernatant. The remaining cells were washed with ice-cold PBS buffer. After centrifugation for 5 min at 500*g* at 4 °C, the supernatant was discarded, and cell pellets were re-suspended in 100 μl of binding buffer containing 1 μl Annexin V-FITC and 5 μl propidium iodide (PI) staining solution at room temperature in the dark for 15 min.

Finally, 400 μl of binding buffer was added before analysis on COULTER^®^ EPICS^®^ XL^™^ flow cytometer (Beckman Coulter Co., France). Analysis of data was performed using WinMDI software (version 2.8).

### In vitro treatment and irradiation of cancer cell lines

The three cancer cell lines were first grown for 24 h in 25 cm^2^ culture flasks at a cell density of 1 × 10^6^ under hypoxia, then treated with both the IC_50_ concentration (obtained from cytotoxicity MTT test) of the tested chemotherapeutic agents, then irradiated with gamma ray doses of 5 and 10 Gy using a ^137^Cs unit station (0.41 Gy/min, dose rate) (Gamma-cell 40, Canada) under hypoxic and normoxic conditions.

After 24 h of recovery, cells at a density of approximately 2 × 10^6^ were lysed on ice with 200 µl of cold lysis buffer that consists of 20 mM Tris–HCl pH 7.4 containing 137 mM NaCl_2_, 2 mM EDTA, and a 1% Triton X-100. Lysates were transferred to Eppendorf tubes, incubated for 15 min at 4 °C, and centrifuged for 30 min at 4500 rpm at 4 °C. The supernatant was collected and rapidly frozen and stored at − 20 °C for future detection of HIF-1α protein concentration using ELISA.

### Measurement of HIF-1α level using enzyme-linked immunosorbent assay (ELISA)

**Fig. 2:**
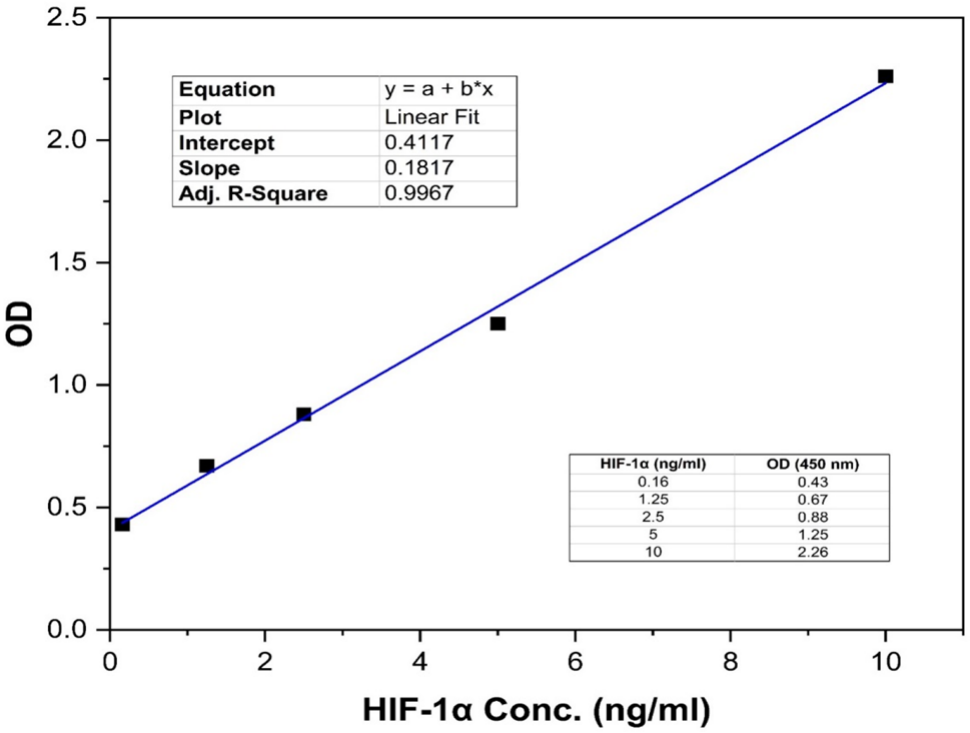
Standard curve for HIF-1α protein. The optical density at 450 nm is plotted versus physiological wide range of HIF-1α concentration. A very strong correlation was found that provided opportunities for accurate determination of HIF-1α in the treated samples

HIF-1α protein levels were examined as a marker of hypoxia and measured using a commercial human HIF-1α ELISA Kit-96 T (Wuhan Fine Biotech Co., China), according to the manufacturers’ instructions. Figure 2 shows the standard curve of the HIF-1α protein.

In the three above major steps including cell toxicity assay, flow cytometry as well as gamma irradiation, the comparison was performed taking the cells grown in normoxia conditions as reference keeping all experimental conditions as similar as possible. This has been widely used in the literature especially in experimental work looking at the effect of oxygen insufficiency on tumor cells while rendering normoxia as control (Bowyer et al. 2017; Dubbelboer et al. 2019; Saggar and Tannock 2015).

## Results

### Cell viability assessment

The drug sensitivity as assessed by the MTT assay under normoxic (20% O_2_) and hypoxic (1% O_2_) conditions showed that the relative effect of hypoxia on the IC_50_ was cell line-dependent.

**Fig. 3:**
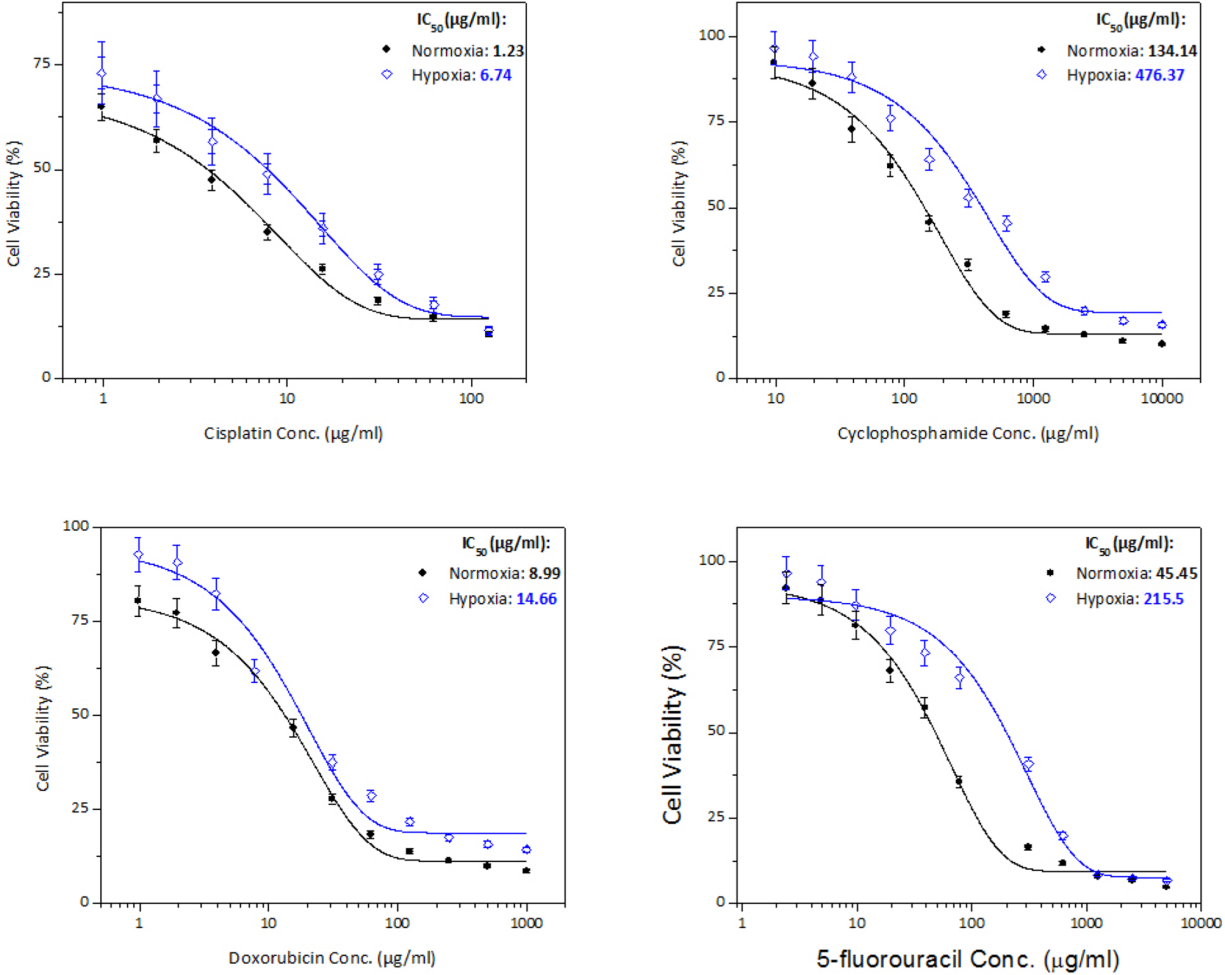
Cell viability of PC3 cell line treated in vitro for 48 h with cisplatin (**a**), cyclophosphamide (**b**), doxorubicin (**c**), and 5-fluorouracil (**d**) under normoxic and hypoxic conditions. Data expressed as percent to control. Error bars represent standard deviations

In all chemotherapeutic agents used on PC3 cells, the IC_50_ was higher when cells were under hypoxic conditions than normal conditions, as shown in Fig. 3 and Table 1. The graphs show the mean absorbance expressed as % of the untreated controls against log concentration of cisplatin, cyclophosphamide, doxorubicin, or 5-fluorouracil. The sigmoid pattern response of cell chemosensitivity was apparent in all drugs used with PC3 cells.

**Table 1:** IC_50_ doses (µg/ml) of chemotherapeutic agents used in the treatment of the different cancer cell lines under normoxic (N) and hypoxic (H) conditions

Drug	PC3 cell line		HepG2 cell line		MCF-7 cell line	
	N	H	N	H	N	H
Cisplatin	1.23	6.74	2.34	8.76	4.82	11.71
Cyclophosphamide	134.14	476.37	275.13	361.46	350.61	500.69
Doxorubicin	8.99	14.66	0.39	4.10	3.30	5.17
5-fluorouracil	45.45	215.50	155.28	236.67	59.28	164.13

*Each value was obtained from 3 readings

**Fig. 4:**
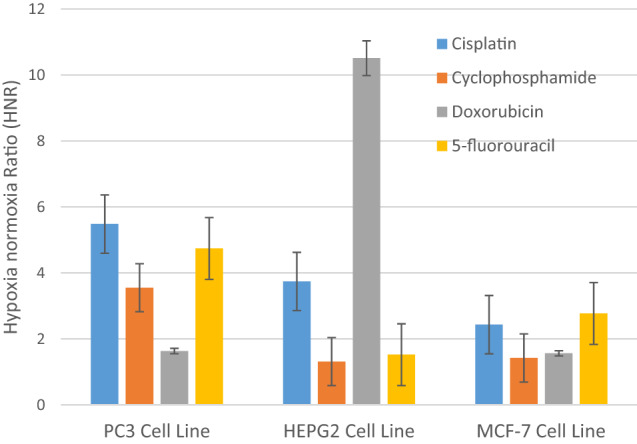
HNR of cancer cell lines PC3, HepG2, and MCF-7 in response to treatment with the different chemotherapeutic agents including cisplatin, cyclophosphamide, doxorubicin and 5-flurouracil. Error bars represent standard deviations

The hypoxia/normoxia ratio (HNR) obtained for PC3 cells is presented graphically in Fig. 4. A similar pattern of response was observed in all chemotherapeutic agents such that the HNR was always greater than one (i.e., HNR > 1). A closer look into Fig. 4 indicated that the HNR was highest in cisplatin, followed by 5-fluorouracil, cyclophosphamide, and doxorubicin, respectively.

**Fig. 5:**
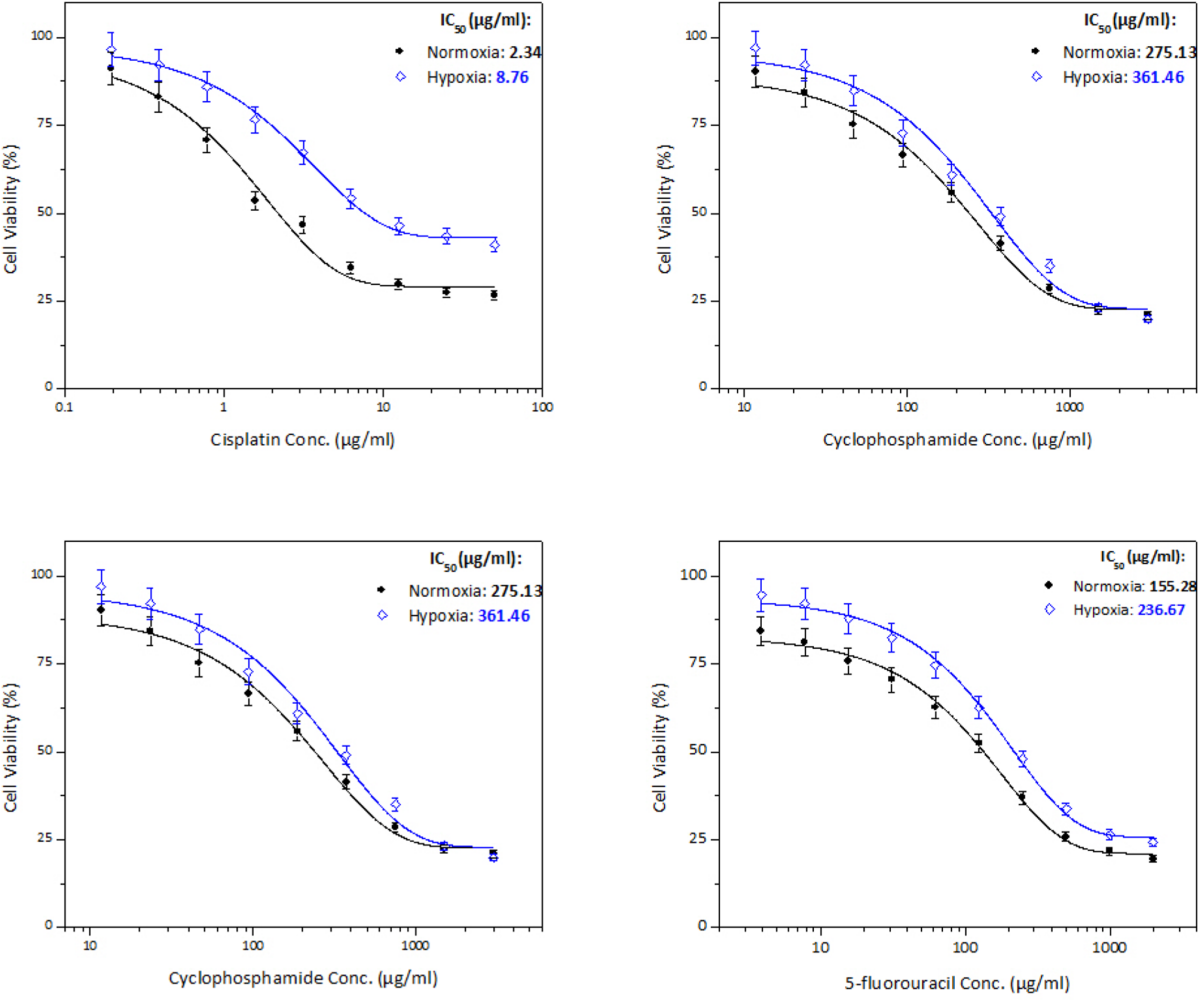
Cell viability of HepG2 cell line treated in vitro for 48 h with cisplatin (**a**), cyclophosphamide (**b**), doxorubicin (**c**), and 5-fluorouracil (**d**) under normoxic and hypoxic conditions. Error bars represent standard deviations

Similar to PC3 cells, the IC_50_ was higher in all drugs used with HepG2 cells when the cells were under hypoxic conditions compared with normoxia, Fig. 5 and Table 1.

As shown in Fig. 4, the HNR was decreased in the order doxorubicin > cisplatin > 5-fluorouracil > cyclophosphamide. The highest HNR of HepG2 cells was about 11-fold when using doxorubicin, while the lowest HNR was obtained when treating with cyclophosphamide at almost slight increase of drug concentration (1.3-fold).

**Fig. 6:**
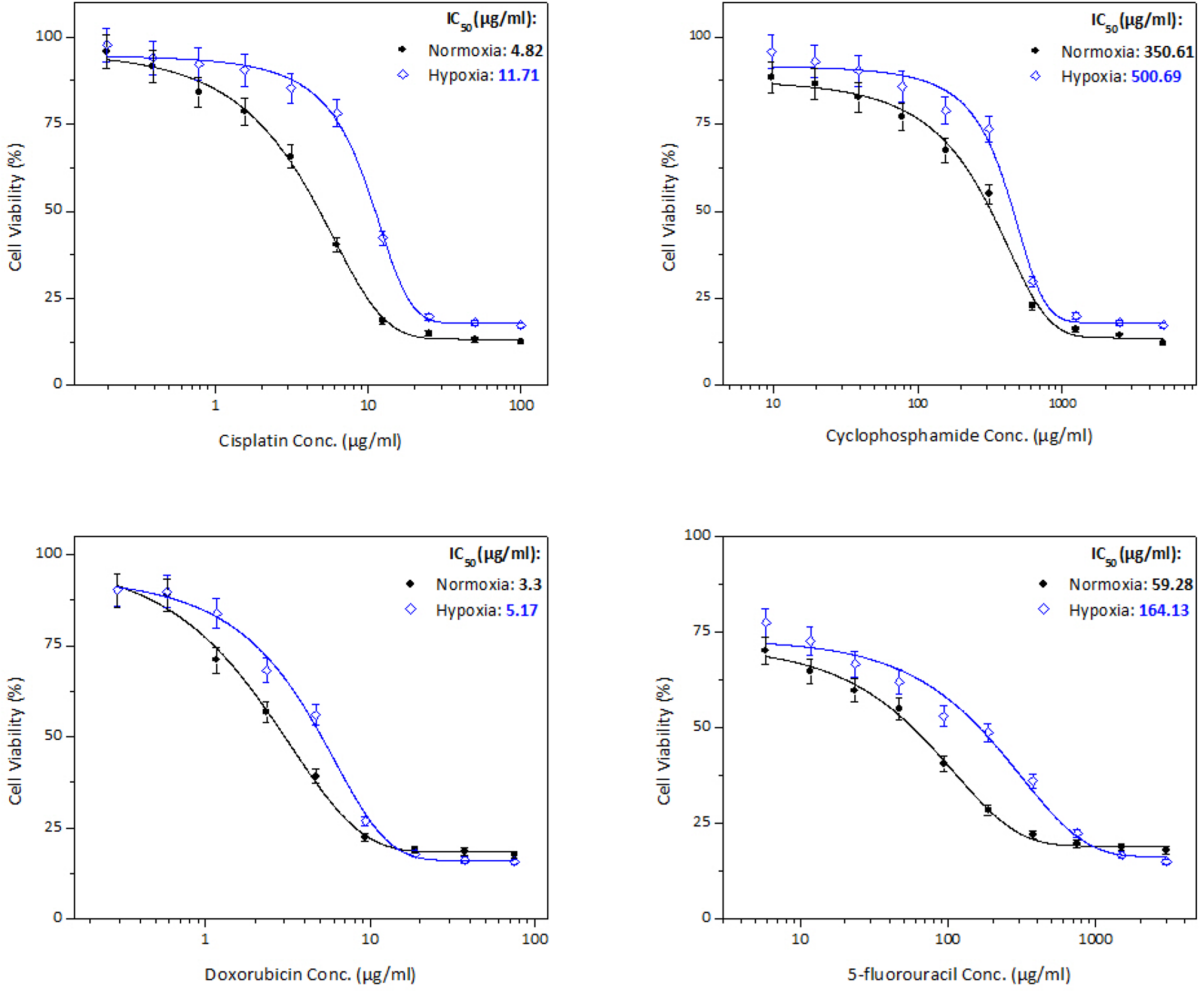
Cell viability of MCF-7 cell line treated in vitro for 48 h with cisplatin (**a**), cyclophosphamide (**b**), doxorubicin (**c**), and 5-fluorouracil (**d**) under normoxic and hypoxic conditions. Error bars represent standard deviations

Similar to PC3 and HepG2 cells, all chemotherapeutic agents used with MCF-7 cells revealed that the IC_50_ was higher when cells were under hypoxic in comparison to normoxic conditions, as shown in Fig. 6 and Table 1. In contrast to the previous two cell lines, the HNR was increasingly higher in this order 5-fluorouracil > cisplatin > doxorubicin > cyclophosphamide, Fig. 4. Moreover, the highest HNR of MCF-7 cells was around threefold when cells were treated with 5-fluorouracil, whereas it was minimum around onefold as shown with cyclophosphamide.

### Flow cytometric data analysis

**Fig. 7:**
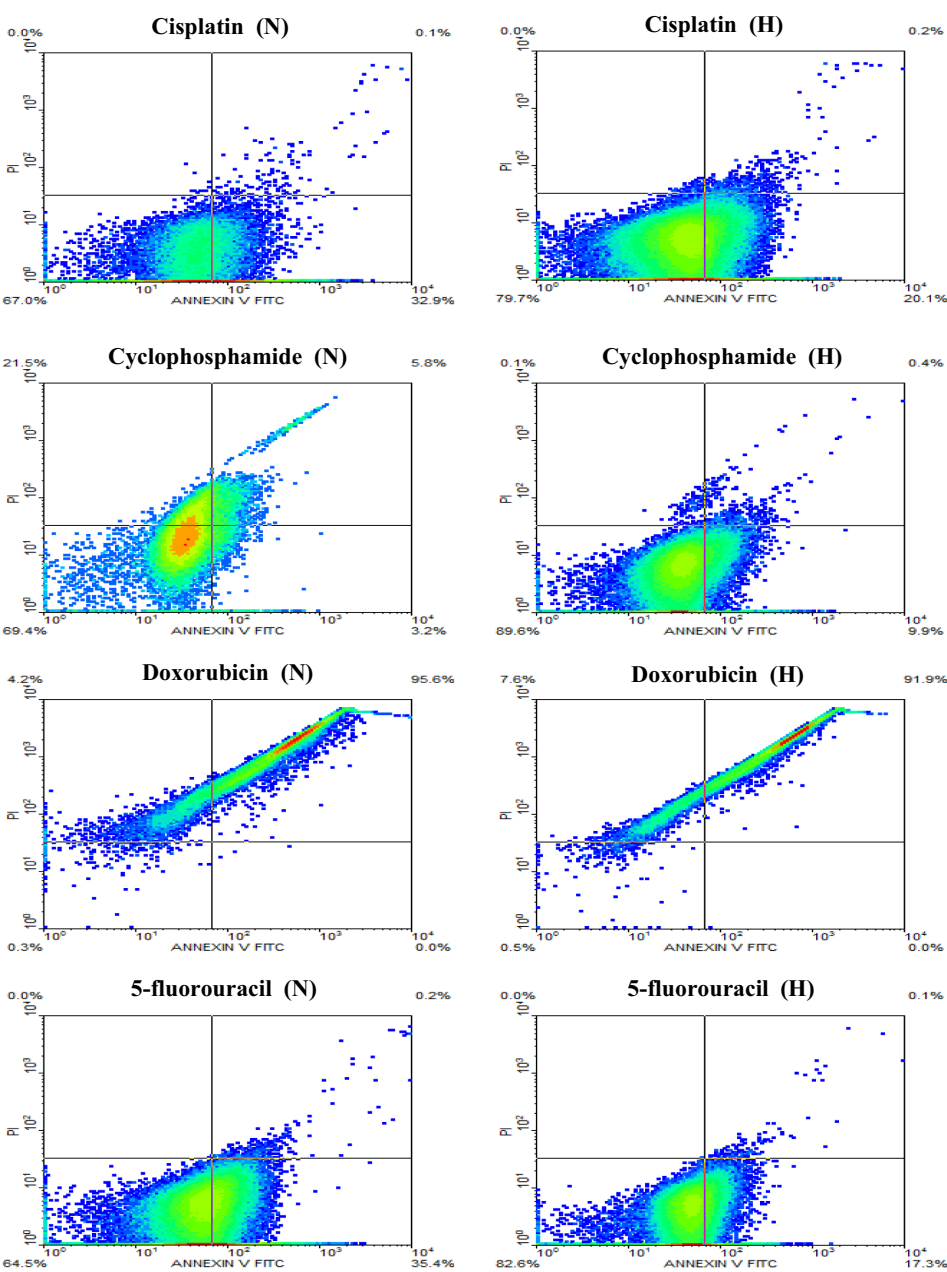
Flow cytometric analysis of PC3 cells treated with the IC_50_ concentration of cisplatin, cyclophosphamide, doxorubicin, and 5-fluorouracil for 24 h under normoxic (N) and hypoxic (H) conditions

**Fig. 8:**
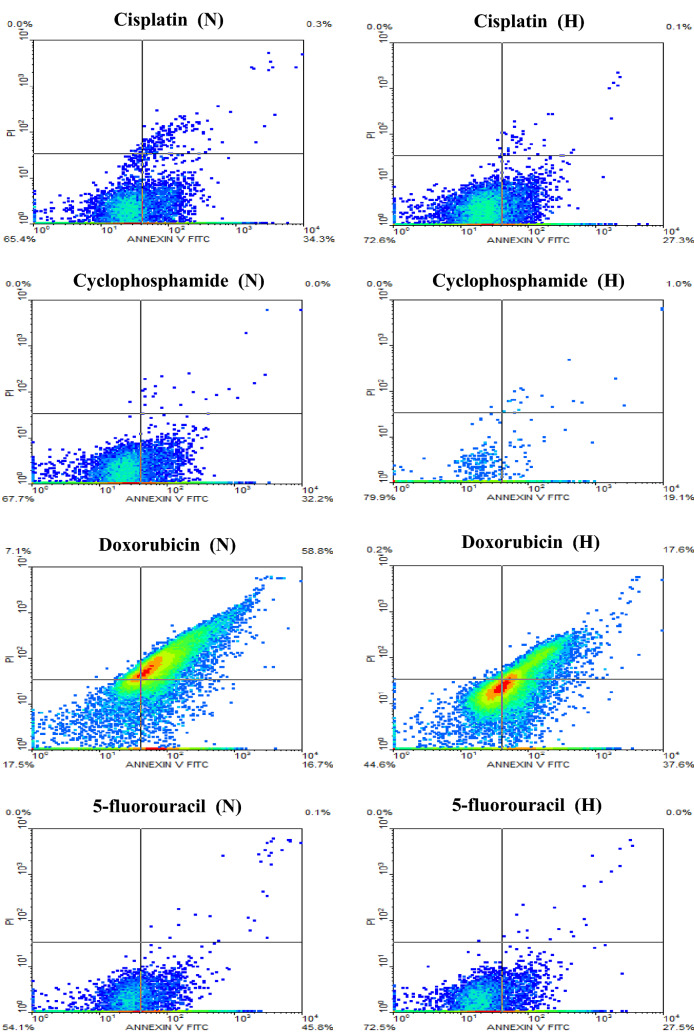
Flow cytometric analysis of HepG2 cells treated with the IC50 concentration of cisplatin, cyclophosphamide, doxorubicin, and 5-fluorouracil for 24 h under normoxic (N) and hypoxic (H) conditions

**Fig. 9:**
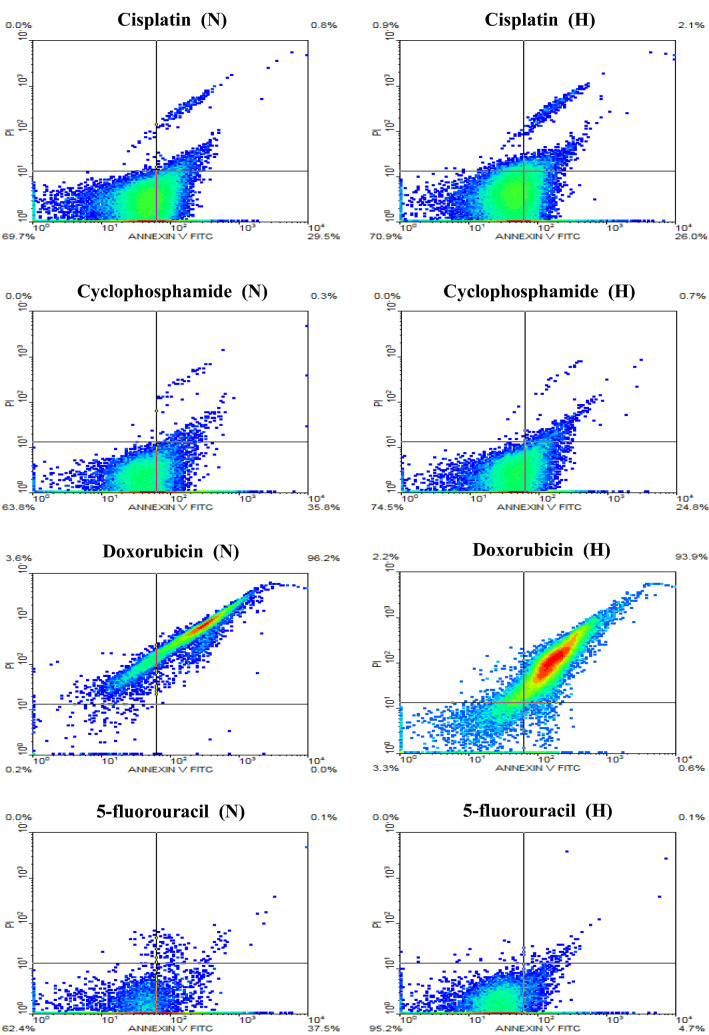
Flow cytometric analysis of MCF-7 cells treated with the IC_50_ concentration of cisplatin, cyclophosphamide, doxorubicin, and 5-fluorouracil for 24 h under normoxic (N) and hypoxic (H) conditions

Flow cytometry was used to verify the results obtained from the colorimetric MTT assay to ensure the induction of cellular apoptosis. In all cancer cell lines, the flow cytometric results confirmed that all chemotherapeutic agents were variably less effective in hypoxia than normoxia. The majority of results obtained were in the direction of reduced cell apoptosis in hypoxia than normoxia when the cancer cells were treated with the different chemotherapeutic compounds. Figures 7, 8 and 9 describe the data of the flow cytometry of investigated cancer cell lines versus the four chemotherapeutic agents. Tables 2, 3 and 4 summarize the results of total apoptotic cell populations (i.e., Q_2_ + Q_4_) as a new index to describe in part the clinical environment where hypoxic and normoxic cells are often in close neighboring relationships.

**Table 2:** Results of flow cytometry of PC3 cells treated with the chemotherapeutic agents under normoxic and hypoxic conditions

Drug	Late apoptosis Q_2_%		Early apoptosis Q_4_%		Q_2_ + Q_4_ %	
	N	H	N	H	N	H
Cisplatin	0.1	0.2	32.9	20.1	33.0	20.3
Cyclophosphamide	5.8	0.4	3.2	9.9	9.0	10.3
Doxorubicin	95.6	91.9	0.0	0.0	95.6	91.9
5-fluorouracil	0.2	0.1	35.4	17.3	35.6	17.4

**Table 3:** Summary of the flow cytometry data of HepG2 cells treated with the chemotherapeutic agents under normoxic and hypoxic conditions

Drug	Late apoptosis Q_2_%		Early apoptosis Q_4_%		Q_2_ + Q_4_ %	
	N	H	N	H	N	H
Cisplatin	0.3	0.1	34.3	27.3	34.6	27.4
Cyclophosphamide	0.0	1.0	32.3	19.1	32.3	20.1
Doxorubicin	58.8	17.6	16.7	37.6	75.5	55.2
5-fluorouracil	0.1	0.0	45.8	27.5	45.9	27.5

**Table 4:** Results of flow cytometry of MCF-7 cells treated with the chemotherapeutic agents under normoxic and hypoxic conditions

Drug	Late apoptosis Q_2_%		Early apoptosis Q_4_%		Q_2_ + Q_4_ %	
	N	H	N	H	N	H
Cisplatin	0.8	2.1	29.5	26.0	30.3	28.1
Cyclophosphamide	0.3	0.7	35.8	24.8	36.1	25.5
Doxorubicin	96.2	93.9	0.0	0.6	96.2	94.5
5-fluorouracil	0.1	0.1	37.5	4.7	37.6	4.8

Annexin V-FITC/PI analysis showed that the chemotherapeutic agents had induced variable cell populations at early and late apoptosis in PC3 cells, Fig. 7. Under hypoxic conditions, the induction of both early and late apoptosis was the highest when the PC3 cells were treated with doxorubicin while being the lowest with cyclophosphamide treatment, Table 2.

Annexin V-FITC/PI analysis revealed that chemotherapeutic agents also induced early and late apoptosis of variable cell populations in HepG2 cells, Fig. 8. Similar to the findings of PC3, the induction of both early and late apoptosis was relatively higher in all treatments administered to HepG2 cells when cells were in hypoxic conditions using doxorubicin while being lower with cyclophosphamide, Table 3.

The analysis of Annexin V-FITC/PI showed that the different chemotherapeutic agents had induced early and late apoptosis of variable cell populations in MCF-7 cells, Fig. 9. In all treatments applied to MCF-7 cells, the induction of both early and late apoptosis was relatively higher when cells were under hypoxic conditions using doxorubicin, while 5-fluorouracil was the lowest, Table 4.

### Quantification of HIF-1α levels using ELISA

The combined chemoradiotherapy in case of PC3 cancer cell line treated with all chemotherapeutic agents showed a reduction of HIF-1α level when the radiation dose has been doubled (i.e., from 5 to 10 Gy), Table 5 summarizes the percent reduction performed of HIF due to cell treatment with combined chemoradiotherapy for all cell lines under 5 and 10 Gy gamma exposure The maximum reduction of HIF-1α level (with respect to hypoxic cells not irradiated) was obtained after treatment of PC3 cells with doxorubicin in addition to radiation doses of 5 and 10 Gy while cyclophosphamide achieved minimal effect in reducing the HIF-1α level in the presence of gamma irradiation, Table 5.

**Table 5:** Percent reduction of HIF-1α level produced by all cancer cell lines treated with the four chemotherapeutic agents and irradiated with 5 or 10 Gy radiation doses under hypoxic conditions

Drug	PC3 cell line		HepG2 cell line		MCF-7 cell line	
	5 Gy %	10 Gy %	5 Gy %	10 Gy %	5 Gy %	10 Gy %
Cisplatin	57.6	63.7	73.4	84.0	62.1	75.5
Cyclophosphamide	12.9	19.1	45.2	63.3	60.6	67.6
Doxorubicin	69.2	83.6	85.6	93.8	81.0	95.5
5-fluorouracil	19.6	62.3	77.8	92.4	9.5	51.7

*Each sample was measured in duplicate

HepG2 cancer cell line treated with all chemotherapeutic agents showed a reduction in the level of HIF-1α when the radiation dose was doubled (i.e., from 5 to 10 Gy). The greatest reduction of the HIF-1α level was achieved with doxorubicin in 10 Gy radiation dose, whereas cyclophosphamide had minimal effect in reducing the HIF-1α level with the radiation doses of 5 and 10 Gy.

In case of MCF-7 cancer cell line treated with all chemotherapeutic agents, the combined chemoradiotherapy also showed a reduction of HIF-1α level when the radiation dose has been doubled (i.e., from 5 to 10 Gy). The maximum reduction of HIF-1α level was observed with doxorubicin in both 5 and 10 Gy radiation doses, whereas 5-fluorouracil achieved minimal effect in reducing the HIF-1α level with 5 Gy radiation dose.

## Discussion

Hypoxic tumors remain one of the challenges for chemotherapy and radiotherapy treatment regimes. This is in great part due to the fact that hypoxia creates a state of treatment resistance that potentially reduces drug efficacy and treatment success (McKenna et al. 2018). Our approach was to characterize the response of different cancer cell lines using different chemotherapeutic agents and then evaluate the impact of combined chemoradiotherapy on the levels of HIF-1α on the same cells under hypoxia taking the normoxic conditions as reference.

Several studies have shown that tumor cells are capable of developing adaptive resistance under stress conditions such as hypoxia which interferes with the mechanisms of action for most cancer chemotherapeutic drugs (Davou et al. 2019) leaving the normal cells more vulnerable to the cytotoxic effects of these drugs.

The oxygen concentration in human tumors widely varies and the level of hypoxia seems to be tumor stage- and size-independent. Radiotherapy and conventional chemotherapies are often less effective in oxygen-depressed cells. Therefore, it is of great importance to make use of oxygen deficiency and find drugs that are more effective in hypoxic tumor cells (Strese et al. 2013).

MTT assay was used to evaluate the anticancer effects of different chemotherapeutic agents on PC3, HepG2, and MCF-7 cancer cells. Exposure of cancer cell lines to 24 h treatment of chemotherapeutic agents might not be long enough to see a reduction in cell viability using the MTT assay, and therefore, a later time point of 48 h could also be investigated (Yu et al. 2015). However, an early recording of the drug potency might indicate its effectiveness since a longer time might equalize the effect of different agents.

In all cancer cell lines, exposure to cytotoxic agents under hypoxic conditions led to significant resistance to all chemotherapeutic agents represented by an increased drug concentration (higher IC_50_) compared to those under normoxic conditions.

Results showed also that when PC3 prostate cancer cells were exposed to hypoxic conditions, they developed resistance to cytotoxic drug cisplatin (i.e. HNR = 5.48) and lower resistance to cytotoxic drug doxorubicin (i.e. HNR = 1.63), whereas hypoxic HepG2 liver cancer cells had more resistance to doxorubicin (i.e. HNR = 10.51) but remarkably lower resistance to drug cyclophosphamide (i.e. HNR = 1.31). MCF-7 breast cancer cells had greater resistance to cytotoxic drug 5-fluorouracil (i.e. HNR = 2.77) and lower resistance to cytotoxic drug cyclophosphamide (i.e. HNR = 1.42).

This signifies that the response of cells to chemotherapeutic agents under normoxic and hypoxic conditions is variable, depending on the cell line and type of chemotherapy. Similar findings were reported with HepG2 when treated with doxorubicin showing more resistance in hypoxia than normoxia after 24 h incubation (Bowyer et al. 2017). On the other hand, chemotherapeutic drugs such as doxorubicin upregulate HIF-1α (Cao et al. 2013) providing a feedback loop amplifying drug resistance responses in tumors. Cisplatin chemo-resistance has been an intriguing issue during hypoxia, and this observation has also been made in the current study as the cisplatin H/N ratio was among the highest across the four drugs in PC3 and comparable with 5-fluorouracil in MCF7 and second after doxorubicin in HepG2 cell lines. Moreover, HepG2 and other liver cell lines showed better response in treatment with cisplatin in normoxia despite the improved cell viability under hypoxia conditions (Chi et al. 2009).

The observed tolerance to doxorubicin was significantly different between the cell lines being higher with HepG2 cells, whereas lowest tolerance with MCF-7 cells. This has been reported not only in different cancers, but also in cell lines of the same type of cancer, for example, liver carcinoma (Dubbelboer et al. 2019). It was shown that hypoxia could interfere with chemotherapeutic treatment and resistance of doxorubicin producing different profiles of protein expression for human-derived liver cancer cells (Dubbelboer et al. 2019).

Highly but variable significant resistance to cisplatin, cyclophosphamide, doxorubicin, and 5-fluorouracil in hypoxia was observed in all the three cancer cell lines, consistent with previous data showing hypoxia-induced resistance to cisplatin and doxorubicin among others in a variety of different tumor types (Adamski et al. 2013).

In all cancer cell lines exposed to various chemotherapeutic agents under hypoxia, apoptosis was generally reduced as displayed by flow cytometry results. However, this reduction was variable being very low in PC3 cells treated with cyclophosphamide and MCF7 treated with cisplatin and doxorubicin. The highest reduction was noted in MCF-7 cells when treated with 5-fluorouracil and in PC3 cells when treated with cisplatin and 5-fluorouracil. This finding confirms the complex nature of hypoxia and its relative impact on different cell lines consistent with previous reports (Blancher et al. 2000; Dubbelboer et al. 2019; Strese et al. 2013).

The difference in the viability of cells after treatment with chemotherapeutic agents is reflected in their differential expression of the apoptotic marker Annexin V.

Inspecting the flow cytometry results, hypoxic cells showed lower levels of total apoptotic cell populations (i.e., Q_2_ + Q_4_) after 24 h of treatment with different chemotherapeutic agents compared to substantially higher levels of total populations of an apoptotic cell under normoxia, certainly, the hypoxia cells still have great resistance to chemotherapy compared to normoxic cells. The doxorubicin drug was obviously a more potent treatment for all cancer cell lines studied under hypoxia.

The combined chemoradiotherapy treatment modalities were administered to prevent treatment resistance due to the higher expression of HIF-1α under hypoxia compared to normoxic conditions. It was reported that in prostate patients who were treated with radiotherapy or radical prostatectomy had a reduced time to disease progression in association with increased HIF-1α levels (Vergis et al. 2008). In patients with hepatocellular carcinoma, the increased HIF-1α was associated with lymph node invasion and patient mortality (Xie et al. 2008).

The ELISA test in the present study allowed for in vitro quantitation of the HIF-1α protein. A multitude of findings showed that as a key regulator of hypoxia adaptive response, the expression level of HIF-1α was closely associated with the type of the cell line, chemotherapeutic agent, as well as a level of gamma irradiation. Under hypoxic conditions, suppression of HIF-1α expression can lead to changes in tumor cell proliferation, invasion, metastasis, and apoptosis. Thus, HIF-1α has gradually become a potential target in the treatment of cancer (Chen et al. 2018).

HIF-1α is a protein that is highly dependent on oxygen content. The presence or absence of oxygen also controls the levels of this protein. In normoxia conditions where there is a sufficient amount of oxygen, HIF-1α is degraded by the complex proteasome, whereas in hypoxic conditions, HIF-1α undergoes stabilization by forming a heterodimer with HIF-1β and then translocates to the nucleus and binds to the gene promoter hypoxia response element (HRE) target (Herawati et al. 2017).

Doxorubicin is one of the most effective antitumor drugs against solid tumors, including breast cancer, but the clinical usage has been limited due to its low bioavailability and severe side effects, such as cardiotoxicity (Gomari et al. 2019). It has many mechanisms of action involving intercalation within DNA base pairs resulting in DNA strand breaks and inhibition of both DNA and RNA synthesis, generating the formation of reactive oxygen species (ROS) and oxidative stress (Korga et al. 2019). Doxorubicin might also serve to inhibit the binding of HIF-1 to the promoter region of genes that respond to low oxygen levels (Lee et al. 2009).

A mechanism by which ionizing radiation exerts its therapeutic effects is by inducing single- and double-strand DNA breaks and ROS. The formation of ROS is dependent on the availability of oxygen. Further, oxygen radiosensitizes cells by stabilizing DNA lesions (Huerta et al. 2009). Radiolysis of water provides the reactive free radicals that damage the DNA (Huerta et al. 2009). Thus, hypoxic tissue responds poorly to ionizing radiation treatment.

It is important to note that HIF-1α activity varies between different cancer cell lines under the same level of hypoxia (Dubbelboer et al. 2019). The amount of HIF-1α protein detected with the ELISA in all cancer cell lines in the control normoxia was relatively low compared to hypoxic conditions. A general but expected observation was that the effect of 10 Gy irradiation was relatively higher in reducing the level of HIF-1α concentration in comparison to 5 Gy in all cell lines using the different chemotherapeutic agents. However, this reduction was not consistent in all the cell lines.

The combined chemoradiotherapy approach taken in this study showed that doxorubicin and cisplatin when combined with 5 Gy gamma irradiation produced the maximal effect in comparison to other drugs in reducing HIF-1α. However, increasing the dose to 10 Gy was not associated with a greater increase in reducing HIF-1α levels except 5-fluorouracil where the reduction was threefold. In HepG2 cells, the drugs doxorubicin, 5-fluorouracil, and cisplatin were the most effective in reducing HIF-1α, and again, increasing the dose was not associated with a large increase in reducing HIF-1α levels. In MCF-7 cells, the doxorubicin, cisplatin, and cyclophosphamide were the highest in reducing HIF-1α levels. However, increasing the radiation dose to 10 Gy was only effective in combination with 5-fluorouracil in significantly lowering HIF-1α levels.

These findings demonstrate the necessity to individually optimize radiation dose with chemotherapeutic agents used in particular cancer cells. They also support notion of precision oncology that aims to tailor a given treatment for every individual patient to reach maximal response. Because the chemotherapy drugs can be used in neoadjuvant or adjuvant treatment either with or without radiation therapy, it is interesting to look for drugs that can reduce the HIF at low radiation doses especially in neoadjuvant chemo-radiotherapy. Such drugs could also have desired consequences in reducing side effects of preoperative regimes (Chen et al. 2019; Matuschek et al. 2020).

Chemoradiotherapy can be used to significantly reduce tumor size and shape as well as reduce tumor recurrence. It can also significantly improve overall survival, progression-free survival in comparison to radiation therapy alone in patients with un-resectable stage III non-small cell lung cancer (Hung et al. 2019). The best combination in preoperative phase should employ lower radiation dose and less toxicity with an increase in tumor resection rate and reduced side effects. Therefore, drugs that enhance tumor radiosensitivity and reduce the hypoxic burden would be highly advantageous. This study has focused on elucidating an important aspect which is the reduction of HIF-1α, emphasizing its utility as a concomitant and prognostic biomarker during chemoradiotherapy (Li et al. 2016). Such effect has been revealed in a meta-analysis on head and neck cancer patients who were treated with radiotherapy or chemoradiotherapy, where the hypoxic fraction was found to be independently associated with patients survival (Nordsmark et al. 2005). While the current study has the limitation of being in vitro, we highly recommend dedicated clinical research to further investigate the combinations of chemoradiotherapy and different dosing schemes so that a better clinical inference can be achieved.

This study highlights future research work to understand individual cellular response variation to combined chemoradiotherapy with more focus on cell apoptosis in association with cellular levels of HIF-1α.

## Conclusion

Cancer cells under hypoxia have a diverse response against chemotherapeutic agents. While doxorubicin has its well-known cytotoxic effects, it showed promising results in reducing cell survival and capabilities of initiating cell apoptosis in cell hypoxia. The hypoxic PC3 cells have greater resistance to cytotoxic drug cisplatin. HepG2 cells have further resistance to cytotoxic drug doxorubicin while MCF-7 cells have greater resistance to cytotoxic drug 5-fluorouracil verifying that the response of cells to chemotherapeutic agents under normoxic and hypoxic conditions varied depending on the cell type. Furthermore, chemoradiotherapy could result in minimal as well as a high reduction of HIF-1α level based on cell type, type of chemotherapy, and amount of ionizing radiation. This study underlines future research work to optimize chemoradiotherapy regime in individual cancer cell hypoxia.
